# Exploring barriers to SARS-CoV-2 testing uptake in underserved black communities in Louisiana

**DOI:** 10.1002/ajhb.23879

**Published:** 2023-02-20

**Authors:** Peter T. Katzmarzyk, Candice A. Myers, Michelle R. Nelson, Kara D. Denstel, Emily F. Mire, Robert L. Newton, Stephanie T. Broyles, John P. Kirwan

**Affiliations:** 1 Pennington Biomedical Research Center, Baton Rouge, Louisiana, USA; 2 Surgeons Group of Baton Rouge, Our Lady of the Lake Regional Medical Center, Baton Rouge, Louisiana, USA

## Abstract

**Objective:**

To collect qualitative data on approaches that can potentially reduce barriers to, and create strategies for, increasing SARS-CoV-2 testing uptake in underserved Black communities in Louisiana.

**Methods:**

A series of eight focus groups, including 41 participants, were conducted in primarily Black communities. The Nominal Group Technique (NGT) was used to determine perceptions of COVID-19 as a disease, access to testing, and barriers limiting testing uptake.

**Results:**

Common barriers to SARS-CoV-2 testing were identified as lack of transportation, misinformation/lack of information, lack of time/long wait times, fear of the test being uncomfortable and/or testing positive, the cost of testing, and lack of computer/smartphone/internet. The most impactful approaches identified to increase testing uptake included providing testing within the local communities; testing specifically in heavily traveled areas such as supermarkets, churches, schools, and so forth; providing incentives; engaging local celebrities; and providing information to the community through health fairs, or through churches and schools. The strategies that were deemed to be the easiest to implement revolved around communication about testing, with suggested strategies involving churches, local celebrities or expert leaders, social media, text messages, public service announcements, post cards, or putting up signs in neighborhoods. Providing transportation to testing sites, providing incentives, and bringing the testing to neighborhoods and schools were also identified as easy to implement strategies.

**Conclusions:**

Several strategies to increase testing uptake were identified in this population. These strategies need to be tested for effectiveness in real-world settings using experimental and observational study designs.

## 1 | INTRODUCTION

The COVID-19 pandemic has emerged as the greatest public health crisis in the past 100 years. The virulent and contagious SARS-CoV-2 virus spread rapidly across the globe, causing severe illness and widespread social and economic disruption. The severity of symptoms range from mild to severe, and as of January, 2023 more than 6.7 million people worldwide have died from associated complications, including more than 1.1 million people in the US (United States) alone (Johns Hopkins University, 2023). Those with pre-existing conditions such as severe obesity, diabetes, heart disease and hypertension are at increased risk of developing serious COVID-19 complications, including death (Cai et al., 2021; Kastora et al., 2022; Katzmarzyk et al., 2020; Qian et al., 2022; Zhang et al., 2022). Early in the pandemic, health disparities in SARS-CoV-2 infection rates became apparent, with high prevalence rates in Black, Native American, and LatinX communities compared to White communities (Bentley, 2020; Price-Haywood et al., 2020; Richardson et al., 2020; Tai et al., 2021).

There is some evidence that both area-level and individual-level characteristics may play a role in explaining variability in SARs-CoV-2 infection rates. For example, an analysis of SARS-CoV-2 test results in New York City revealed that the proportion of Black residents in a zip code tabulation area (ZCTA) was associated with an increased risk of a positive test (DiMaggio et al., 2020). A study conducted in Louisiana demonstrated a relationship between higher levels of the Area Deprivation Index (ADI), a multidimensional measure of a community’s socioeconomic position, and higher levels of SARS-CoV-2 infection (Madhav et al., 2020). Similarly, residents of Baton Rouge, Louisiana who resided in a high versus mid-level ADI ZCTAs had a higher odds of SARS-CoV-2 infection (Feehan et al., 2021). In that study, individual-level factors such as race and age were also important; after adjustment for ADI, Black and younger residents had a higher odds of infection compared to White and older residents, respectively (Feehan et al., 2021).

Viral and antibody testing for SARS-CoV-2 is a key element in the public health strategy to mitigate viral spread (Studdert & Hall, 2020). Few studies have evaluated the association between SARS-CoV-2 testing per se and infection control, however, higher testing coverage (tests per confirmed case) has been shown to be associated with lower mortality and case-mortality rates (Wei et al., 2020). Furthermore, a study of data from 173 countries reported that higher testing intensity was a highly significant predictor of reduction in COVID-19 reproduction (transmissibility) across 173 countries early in the pandemic (Rannan-Eliya et al., 2021). On the other hand, mass random testing of the population alone may not be an effective strategy to address pandemics; one study estimated a 2% mean reduction is transmission of SARS-CoV-2 using this approach (Kucharski et al., 2020) However, testing is a key element of the test-trace-isolate strategy (where identified cases are asked to isolate and their contacts are traced and asked to isolate) that was used globally in an attempt to prevent the spread of SARS-CoV-2 (Grantz et al., 2021). Modeling studies have shown that combining self-isolation with testing/contact tracing can significantly reduce the transmission of SARS-CoV-2 (Aleta et al., 2020; Kucharski et al., 2020).

In addition to concerns regarding the contribution of health disparities to the risk of COVID-19 infection and complications, there were concerns about health disparities in access to testing and testing uptake, especially early in the pandemic (Dodds & Fakoya, 2020). Few studies have directly addressed this issue; however, data from New York City indicated a positive association between the proportion of White residents residing in a zip code and the number of total tests performed (Lieberman-Cribbin et al., 2020). Similarly, a statewide study from Massachusetts found that increasing levels of the Social Vulnerability Index (SVI) were associated with increasing testing gaps (Dryden-Peterson et al., 2021). Another study suggested that racial disparities may also exist in the context under which testing occurs: Black adults were more often tested in hospitals, and were less likely to be tested in ambulatory settings compared to other racial/ethnic groups (Azar et al., 2020).

In addition to “access” per se, race differences in testing rates may also be related to issues of stigma (i.e., people who test positively may be viewed differently by their community) (Baldassare et al., 2020), financial disincentives to receiving a positive test result (i.e., cannot return to work), and mistrust associated with test locations and the organizations conducting the testing among minoritized populations (distrust of the medical system and researchers) (Corbie-Smith et al., 1999; Corbie-Smith et al., 2002; Powell et al., 2019). Thus, there is a need to identify strategies to increase the reach and awareness of SARS-CoV-2 testing and therapeutics, especially in underserved populations.

The purpose of this study was to collect qualitative data on community-engaged approaches that can potentially reduce barriers to, and create strategies for, increasing SARS-CoV-2 testing uptake in underserved Black communities in Louisiana.

## 2 | METHODS

Participants were recruited from five zip codes in Baton Rouge, Louisiana, U.S. with a high proportion (≥80%) of Black Americans. These communities were selected based on demographic data obtained from the Census, as well as their geographic proximity to the Pennington Biomedical Research Center. The total adult population in these five zip codes is 80 140, and the population is 91.3% Black (range 81%–96%), and 30.6% (range 12%–39.4%) of the population lives under the poverty line (Census Reporter, 2023). Participants were recruited through word of mouth, distributing flyers in the community, and by contacting participants from our previous studies who had indicated they were willing to be re-contacted for future research. Inclusion criteria included being 18 years of age or older, able to understand and speak English, and able to provide informed consent. Demographic information was collected using an on-line questionnaire administered via REDCap (Harris et al., 2009) that included questions about age, race/ethnicity, sex, education, and annual household income.

The Nominal Group Technique (NGT) was used to determine perceptions of COVID-19 as a disease, access to SARS-CoV-2 testing, and barriers limiting testing uptake. NGT, a brainstorming tool for highly structured small group discussions, is used to elicit and prioritize a list of answers to specific questions (Castiglioni et al., 2008; Crenshaw et al., 2011; Pena et al., 2012; Van de Ven & Delbecq, 1972). The multistep NGT design systematically stimulates meaningful interpersonal statements among participants by gathering equally weighted responses to a specific question, and these statements tend to be a valid representation of group views (Jefferson et al., 2010; Miller et al., 2000). The NGT does not require audio recording and transcription because verbatim responses are usually written on a flipchart, thereby providing a concise summary of the session that is readily available for dissemination. Given the challenges to conducting in-person focus groups during the COVID-19 pandemic, all focus groups were conducted using a virtual meeting platform, and instead of a flipchart, verbatim responses were recorded on a document that was “shared” by a note taker for viewing by all participants. Up to seven participants per group is ideal for NGT sessions (McMillan et al., 2014).

After welcoming, brief introductions, and preliminary probing questions, the facilitator posed a series of questions related to COVID-19 testing in the community, followed by free discussion about each question. These questions related to barriers to getting tested and other factors that prevent community members from getting tested. The specific questions were “What are the main barriers you face to getting tested?” and “What other factors prevent community members from getting tested?”. After the preliminary questions about barriers, the primary question addressed in the focus group was “How can we determine the best community-driven approach to reduce barriers to increasing COVID-19 testing, especially in the Black community?” After listening to the question, each participant generated up to three ideas, which were collated, and duplicates were discarded. The group voted on (1) ideas that would have the greatest impact, and (2) ideas that would be the easiest to implement. The top ranked ideas in each category were generated by discussion and consensus of the group.

All procedures were in accordance with the ethical standards of the responsible committee on human experimentation (institutional and national) and with the Helsinki Declaration of 1975, as revised in 2000. The protocol was approved the Pennington Biomedical Research Center Institutional Review Board, and informed consent was obtained from all participants included in the study.

## 3 | RESULTS

A total of eight focus groups, including 41 participants, were conducted between February 28, 2021 and February 24, 2022. Five focus groups were conducted during February and March 2021 during a period following the early Spring 2021 peak in cases, when SARS-CoV-2 vaccinations were just becoming available. The final three focus groups were conducted in February 2022 following the surge in cases caused by the Omicron variant, which was characterized by a large number of cases but a lower case-mortality rate. Each focus group enrolled between 4 and 6 participants. Descriptive characteristics of the sample are provided in Table 1. The sample was predominantly Black (95.1%), consisted primarily of women (87.8%), and the average age was 58.8 years.

When asked the question “Is COVID-19 a major health concern to you?”, 36 participants responded “yes” (88%), while five participants responded “no” (12%). All 26 (100%) participants in the Spring 2021 focus groups responded “yes”, while 10 (67%) of the 15 participants in the Spring 2022 focus groups responded “yes”.

Table 2 provides the tabulated responses to questions regarding barriers to testing and other factors that prevent community members from being tested. Across all eight focus groups, the most prevalent factors preventing community members from getting tested include lack of transportation (seven groups), misinformation/lack of information (seven groups), lack of time/long wait times (five groups), fear of test/fear of testing positive (three groups), test being uncomfortable (two groups), cost of testing (two groups), and lack of computer/smartphone/internet (two groups).

The top-ranked responses to the question “How can we determine the best community driven approach to reduce barriers to increasing COVID-19 testing, especially in the Black community?” are presented in Table 3 for each focus group. The most common theme for the most “impactful” approach was to provide testing within the local communities, especially for the elderly and disabled/homebound, through the use of mobile units or getting healthcare providers out to the neighborhoods (groups 4, 5, 6, and 7). Along the same lines, two groups suggested providing testing specifically in heavily traveled areas such as Walmart, supermarkets, churches, schools, and so forth. (groups 1 and 2). Three groups suggested that providing incentives would be helpful (groups 4, 7, and 8), while two groups suggested engaging local celebrities to spread the word about testing (groups 7 and 8). Finally, providing information to the community through health fairs, or through churches and schools was also suggested (groups 2 and 3).

The most common theme for strategies that would be “easiest to implement” revolved around communication about testing, with suggested strategies involving churches (groups 3 and 6), social media (groups 1 and 7), or through text messages (group 8), public service announcements (group 2), post cards (group 2) or putting up signs in neighborhoods (group 2). Another suggested strategy related to communication was to engage local celebrities (groups 1, 7 and 8) or expert leaders (group 1) in getting the word out. Other suggestions included providing transportation to testing sites (groups 5 and 6) or providing incentives (groups 4 and 8). Finally, bringing the testing to neighborhoods and schools was also suggested (groups 4 and 7).

## 4 | DISCUSSION

This study provides qualitative data about strategies to improve SARS-CoV-2 testing uptake in underserved Black communities. Common barriers to testing included lack of transportation, misinformation/lack of information, and lack of time/long wait times. Commonly reported strategies with the potential to be impactful revolved around increasing access to testing by bringing the testing to the communities themselves and to the places where most people congregate in the community. The strategies deemed to be easiest to implement revolved mainly around improving communication about the availability of testing.

A rapid scoping review of barriers and strategies to address SARS-CoV-2 testing hesitancy identified 62 studies and gray literature that addressed this topic (Embrett et al., 2022). The most cited barriers to testing included lack of information/misinformation, cost of testing, lack of trust in the healthcare system, availability of testing, and stigma and consequences of testing positive. Identified strategies to increase testing uptake included free testing, promoting awareness of the importance of testing, providing transportation to testing centers, providing several testing options, and offering support for self-isolation. It should be noted that the majority of the literature sources reviewed were opinion pieces or guidelines, rather than experimental evidence (Embrett et al., 2022). The results of our study, conducted in Black urban communities, provide first-hand empirical data that are consistent with primary and secondary source data in the literature, and highlight important strategies that can now be tested using experimental and observational study designs, especially in underserved populations.

The results of the present study are also consistent with three qualitative studies among Black communities. A series of focus groups were conducted among 36 Black Americans living in five rural and urban low-income communities in Alabama (Bateman et al., 2021). Similar to the results reported in the present study, barriers to SARS-CoV-2 testing included misunderstanding, fear, mistrust, testing restrictions and locations of the testing sites. Furthermore, reported strategies to increase testing included providing incentives, clear information from trusted sources, convenient testing locations, and free tests. Another qualitative study among 62 Black Americans from Tennessee identified medical and government mistrust, poor communication, lack of access, stigma associated with positive tests, and costs as major barriers to testing (Kas-Osoka et al., 2022). Additionally, a qualitative study conducted among 26 Black Americans living in Washington, DC identified several barriers to testing, including uncertainty about test accuracy and safety, stigma of positive test results, and concerns about racial bias in health care (Schaffer DeRoo et al., 2021). There is concern that structural racism and disparities in key social determinants of health are contributing to barriers to testing and access to care during the COVID-19 for minoritized populations (Egede et al., 2021). The degree to which the community-based strategies to overcome barriers to testing identified in these studies can overcome structural barriers is not known. Further testing of these strategies using intervention study designs is required.

Five of the focus groups were conducted in February and March of 2021, soon after SARS-CoV-2 vaccines became available, while the final three focus groups were conducted in February of 2022 when vaccines were widely available. While the questions in the focus group script were clearly about testing, the focus group participants were hypersensitive to issues related to the vaccine, and the discussions tended to drift in that direction. For example, when discussing the barriers to testing, 13 of the comments across the eight focus groups were actually about barriers to wearing masks and taking the vaccine, such as lack of education and knowledge about the vaccines, misinformation (i.e., implanting of microchips, etc.), and not knowing the long-term effects of the vaccine. These comments were coded as “off-topic” and are not provided in the tables.

This study has several strengths and limitations. A marked strength of the study is the engagement of a sample of Black Americans from underserved neighborhoods, and the results may be generalizable to other similar populations across the U.S. However, because of the homogenous nature of our sample, the results of this study may not be generalized to other populations. For example, the sample was somewhat older (58.8 years) and consisted primarily of women (87.8%). As described above, this study was conducted during two different phases of the pandemic after vaccines for SARS-CoV-2 became available. It is unclear whether the barriers and strategies identified in this study would be as pertinent to the early stages of an epidemic prior to vaccine availability.

The results of this study provide novel insights into the experiences and opinions of Black community members about the barriers to SARS-CoV-2 testing and potential strategies to improve access and uptake. These results can be used to generate community testing strategies for future epidemics and can also inform the response to other public health emergencies such as natural disasters (i.e., distribution of humanitarian aid, etc.). The testing strategies identified in these focus groups should be rigorously tested for effectiveness using community-based participatory research models, and for generalizability to other populations.

## Figures and Tables

**TABLE 1:** Descriptive demographic characteristics of the study sample.

Age (years)	58.8 (12.8)
Sex (% Female)	36 (87.8%)
Race (% Black)	39 (95.1%)
Hispanic (%)	1 (2.5%)
Household income (*n*, [%])	
Less than $15 000	2 (4.8%)
$15 000-$34 999	12 (29.3%)
$35 000-$74 999	14 (34.1%)
$75 000 and above	4 (9.8%)
Missing	9 (22.0%)
Education (*n*, [%])	
Some high school	1 (2.4%)
High school graduate/GED	3 (7.3%)
Some college/technical/vocational degree	14 (34.1%)
Bachelor’s degree or higher	22 (53.7%)
Missing	1 (2.4%)

*Note*: Results are presented as mean (SD) and n (%).

**TABLE 2:** Barriers to being tested and other factors that prevent community members from being tested.

	Barriers to getting tested	Other factors that prevent community members from getting tested
Group 1	(1) Lack of education and knowledge; (2) Some populations have no concern for healthcare/medicine or hygiene; (3) Lack of knowledge/misinformation	(1) Lack of transportation; (2) Lack of knowledge about where testing sites are; (3) No smartphones, laptops, and internet
Group 2	(1) Lack of transportation; (2) Lack of information; (3) Lack of appointments	(1) Lack of trust; (2) Misinformation through family and friends; (3) Testing times are not convenient
Group 3	(1) Having to travel great distances for a test; (2) Waiting in line	(1) Lack of transportation; (2) Do not feel the need to get tested without symptoms
Group 4	(1) Mental barriers; (2) Long wait time for results; (3) Fear of testing positive; (4) Charges to insurance	(1) Lack of time; (2) Not understanding what the test is; (3) Uncomfortable test (nasal swab)
Group 5	(1) Lack of transportation; (2) Elderly or homebound cannot get out; (3) Fearful of getting tested	(1) Lack of understanding; (2) Fearful—may harm Black people (everyone takes same test); (3) Myths and false information being spread; (4) Will make you sick if tested
Group 6	(1) Long wait times	(1) Fear of test; (2) Lack of computer and internet access; (3) Lack of transportation; (4) Peer allegiance/fake news; (5) Fear of testing positive
Group 7	(1) Uncomfortable test (nasal swab)	(1) Lack of transportation; (2) Misinformation
Group 8	(1) Lack of transportation; (2) Long wait times; (3) Lack of time	(1) Misinformation; (2) Cost of test

**TABLE 3:** How can we determine the best community driven approach to reduce barriers to increasing COVID-19 testing, especially in the Black community?

	Most impactful	Easiest to implement
Group 1	(1) Set up kiosks/tables at Wal-Mart, Asian and Hispanic markets, supermarkets, barbershops, beauty shops, and so forth, and other places where people congregate	(1) Market through social media; (2) Engage local celebrities; (3) Educate and provide trusted information from expert leaders
Group 2	(1) Health fair to distribute information throughout the neighborhood	(1) Put up signs in neighborhoods to let people know where testing sites are; (2) Public service announcements; (3) Post cards
Group 3	(1) Communication through churches and schools, and their involvement with the community; (2) Emergency COVID-19 health alerts on cell phones and TVs	(1) Communication through churches
Group 4	(1) Put testing in heavily traveled areas such as bus stops, churches, schools, and clubs where people congregate; (2) Testing and advertising in residential areas; (3) Partner with local businesses to provide incentives	(1) Testing and advertising in residential areas; (2) Testing in schools; (3) Provide incentives
Group 5	(1) Provide testing for homebound individuals without transportation; (2) Forum for seniors	(1) Provide transportation
Group 6	(1) Get health care providers out to the community, especially to elderly/disabled and explain it to children	(1) Provide transportation; (2) Involve the churches
Group 7	(1) Mobile testing units in communities; (2) Provide incentives; (3) Use social media & celebrities to spread the word	(1) Mobile testing units in communities; (2) Use social media & celebrities to spread the word
Group 8	(1) Engage local celebrities; (2) Provide incentives; (3) Meals	(1) Engage local celebrities; (2) Provide incentives; (3) Text messages

## Data Availability

The data that support the findings of this study are available from the corresponding author upon reasonable request.
